# Martin Leverkus, 1965–2016

**DOI:** 10.1038/cddiscovery.2016.93

**Published:** 2017-01-23

**Authors:** Henning Walczak, Martin R Sprick

**Affiliations:** 1 UCL Cancer Institute, University College London (UCL), London, UK; 2 DKFZ, Heidelberg, Germany


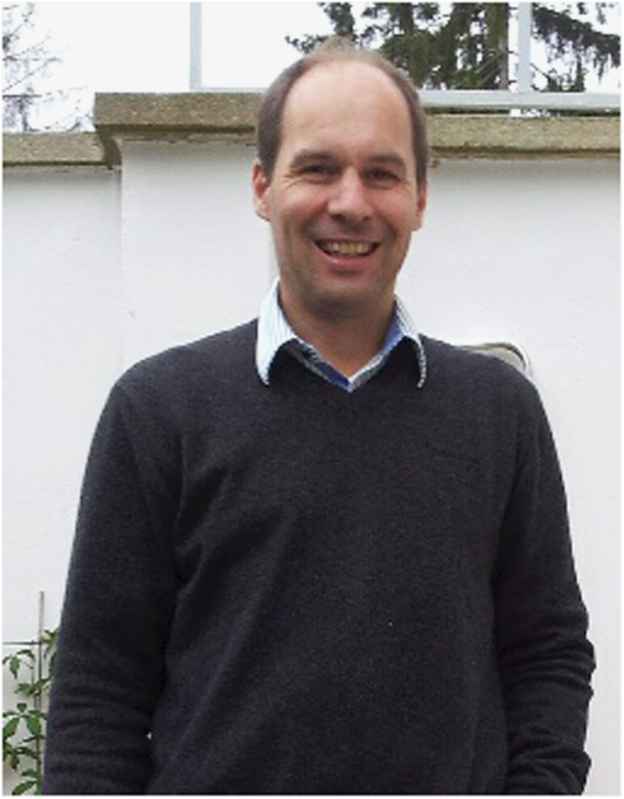


With great sadness we announce the loss of Martin Leverkus who recently died, completely unexpectedly, of acute heart failure. He will be deeply missed by his family, friends, colleagues and everyone in the cell death community.

Martin was originally from Cologne, Germany, where he trained as a physician. After completing medical studies Martin was a resident in the Dermatology Department of the University of Würzburg before pursuing postdoctoral studies in the laboratory of Barbara Gilchrest at Boston University. In 1997 Martin returned as resident in dermatology to the University of Würzburg, where he obtained board certification in Dermatology and Allergology in 2000. After holding positions as Associate Professor in Magdeburg and Mannheim, Germany, in 2015 he was appointed Full Professor and Chair of the Department of Dermatology and Allergology at the University of Aachen. In the same year he also received the Oscar-Gans Award, the most prestigious Prize awarded by the German Society for Dermatology, for outstanding contributions to the field of Experimental Dermatology, specifically mentioning his contribution to the characterization of fundamental mechanisms and processes regulating programmed cell death in the skin.

Martin first developed an interest in the study of cell death during his time as a postdoctoral fellow in Boston, where he discovered the importance of CD95 signalling to UV-induced apoptosis in keratinocytes. Soon thereafter his interest in studying the mechanisms of cell death expanded, leading to deeper understanding of the regulation of death-receptor-induced apoptosis in general with a specific focus on the regulatory proteins cFLIP and the cIAPs in this process. In 2011 his laboratory described the ripoptosome, a complex consisting of RIPK1, FADD and caspase-8, which assembles in response to loss of cIAPs.

Martin was always keen on translating basic discoveries to the clinic. He made major contributions to our current understanding of the aetiology of dermatologic diseases, particularly with regard to the role played by deregulated cell death mechanisms.

Those of us who were privileged to know Martin in the context of his clinical work will have witnessed that he was an exceptionally caring and compassionate physician. For Martin, medicine was his profession and science was his passion. He was always enthusiastic about discussing science from basic aspects to disease implications, covering areas from technical even to philosophical aspects of science and society. Martin managed to combine skills as an excellent practising dermatologist with basic research at an outstanding level, exemplified by his discovery of the ripoptosome. Even with his great success Martin remained modest and self-deprecating.

Apart from science and medicine, Martin also loved skiing and playing tennis. He often gave helpful advice on how to improve one’s skiing skills, as many who visited one of the Keystone conferences on cell death with him can confirm. The Keystone conferences were his favourite meetings and we and the late Jürg Tschopp often arranged to arrive one day earlier to get in an entire day of skiing with him before the meeting started.

From childhood on Martin loved classical music and he was an avid violinist. When his grandmother died he inherited her violin because, as she had once put it, no one had played it more beautifully than Martin. He also loved opera, and it is therefore not surprising that Martin met his wife Charlotte, a mezzo soprano, through his love of music and opera, connecting his first love to the most important one of his life. Together, they were fortunate to add their two wonderful daughters to complete their family.

With Martin Leverkus we all lost a kind and gentle man, a wonderful human being, a great friend, an outstanding scientist and a caring medical doctor. Our thoughts are with his family who mourn the tragic and untimely loss of a wonderful husband, father, brother and son.

